# Comparative in vitro cytotoxicity of free curcumin and a liposomal curcumin formulation on various human cancer cell lines

**DOI:** 10.1038/s41598-026-36607-x

**Published:** 2026-02-11

**Authors:** Said A. Ali, Hagar I. Helmy, Mohamed H. Gaber

**Affiliations:** 1 Biophysics Department, Faculty of Science, Cairo University, Giza, 12613 Egypt; 2 Basic Science Department, School of Engineering, May University in Cairo, Cairo, Egypt

**Keywords:** Drug delivery, Curcumin, Cytotoxicity assay, Liposomes, MCF7ADR, Biochemistry, Biotechnology, Cancer, Drug discovery, Nanoscience and technology

## Abstract

Phospholipids derived from plants are promising for drug delivery applications. This study aimed to develop liposomes from cost-effective, plant-derived soy lecithin to enhance the cytotoxicity of curcumin (CUR) against a panel of human cancer cells. The optimized curcumin-loaded liposomes (CUR-Liposomes) were characterized and exhibited a nanoscale size of 105.7 nm, and a high negative zeta potential of -49.9 mV. The key finding from the in vitro cytotoxicity assay was that CUR-liposomes demonstrated significantly enhanced anticancer activity compared to free CUR, as evidenced by lower half maximal inhibitory concentration (IC50) values across all tested cancer cell lines, including multidrug-resistant MCF-7 breast adenocarcinoma (MCF-7/ADR), colon (Caco-2), lung (A549), prostate (PC3), and pancreatic (PANC-1) cancer cells. Notably, this enhanced cytotoxicity was not observed in normal Vero cells, suggesting a favorable selectivity profile. These findings demonstrate that liposomal encapsulation using plant-derived lipids is a viable strategy to improve the therapeutic efficacy and potential selectivity of CUR for cancer treatment.

## Introduction

Cancer is one of the leading causes of mortality worldwide, and the disease’s death rates remain relatively high even with increased global awareness and the availability of multitargeted treatment options^1,2^. Chemotherapeutic drugs are commonly used to treat cancer; nevertheless, these agents have significant side effects since they are harmful to both tumor and normal cells. Furthermore, most people cannot afford these agents due to their unreasonable cost. Additionally, these substances cannot be utilized to prevent cancer. Thus, searching for cancer therapy approaches that are both more effective and less harmful is at the forefront of the current research. Traditional plant-derived therapies are often free of harmful side effects and are usually inexpensive: CUR is one such drug that is safe, economical, and effective^3^. CUR, the main element in the *Curcuma longa* plant, has gained much interest as an anti-inflammatory, antioxidant, and anticancer agent over the last two decades. CUR’s unique anticancer efficacy is primarily mediated by inducing apoptosis and reducing tumor proliferation and invasion via a range of cellular signaling pathways^4,5^. CUR has been found to have antitumor activity against breast cancer, head and neck squamous cell carcinoma, lung cancer, brain tumors, and prostate cancer. Several studies have demonstrated its ability to target numerous cancer cell lines^5^.

Despite all the benefits listed above, CUR’s low water solubility, which leads to low chemical stability and poor oral bioavailability, limits its applications^6^; an additional obstacle is the limited cellular absorption of CUR. Owing to its hydrophobic nature, CUR tends to penetrate the cell membrane and bind to the fatty acyl chains of the membrane lipids via hydrophobic interactions and hydrogen binding; this leads to a decrease in CUR’s availability within the cytoplasm^5,7^. To overcome all these obstacles and enhance CUR’s overall anticancer effectiveness, various delivery systems, such as liposomes, are used^8,9^.

Liposomes are phospholipid bilayer vesicles that can transport both hydrophobic and hydrophilic pharmaceuticals. Liposomes’ amphiphilic phospholipid bilayer is very similar to the mammalian cell membrane, allowing for efficient interactions between liposomes and cell membranes and, as a result, successful cellular uptake. Combining CUR with liposomes is a valuable strategy to overcome cancer cells’ multidrug resistance. This is because liposomes can increase CUR’s water solubility and bioavailability^10^, and CUR itself can overcome drug resistance by controlling multidrug-resistant cancer cells’ signaling pathways, decreasing the expression of proteins linked to drug resistance, reversing mechanisms of multidrug resistance, and increasing the sensitivity of multidrug-resistant cells^11^.

Phospholipid-based formulations have gained significant attention in recent years due to their safety, stability, and suitability for pharmaceutical applications. Soy lecithin, in particular, is considered an economical and widely available phospholipid source that can be used for cost-effective liposome preparation. Several previous studies have investigated liposomal formulations of CUR; however, many of these reports evaluated their cytotoxicity in only one or two cancer cell lines, which restricts understanding of how different tumor types may respond to free versus encapsulated CUR. To address this limitation in scope, the present study examines the effect of free CUR and CUR-loaded soy lecithin liposomes across a broader panel of human cancer cell lines, including MCF-7/ADR, A549, Caco-2, PANC-1, and PC-3, in addition to a normal Vero cell line. The liposomal formulation was prepared using the thin-film hydration method and characterized by particle size distribution, zeta potential, transmission electron microscopy (TEM), Fourier-transform infrared spectroscopy (FTIR), and encapsulation efficiency. CUR in both forms was then tested on all selected cell lines to obtain a wider overview of its cytotoxic profile. This broader cell-line panel provides a more informative and translational perspective compared with earlier studies that relied on a limited number of cancer models.

## Materials and methods

This study employed various materials, including CUR (> 95% purity; molecular weight 368.38 g/mol), purchased from Loba Chemie (Mumbai, India). Soy lecithin (SL) powder (molecular weight 643.97 g/mol) was obtained from bulk-supplements.com (Henderson, NV, USA). Cholesterol (CHOL; molecular weight 386.65 g/mol; and melting point 148–150 °C) was purchased from Advent Chembio Pvt. Ltd. (India). Chloroform (trichloromethane), ethanol (99%), dimethyl sulfoxide (DMSO), and MTT (3-(4,5-dimethylthiazolyl)-2,5-diphenyl-tetrazolium bromide) were obtained from Alfa Chemical Stores (Egypt). Distilled water and saline solution were used throughout the experimental procedures. A549, Caco-2, PANC-1, PC-3, MCF-7/ADR, and Vero cells were obtained from the Egyptian Holding Company for Biopharmaceuticals and Vaccines Production (Vacsera, Cairo, Egypt).

### Preparation of CUR solution

A solution was made by dissolving CUR in dimethyl sulfoxide (DMSO) to achieve a 1.2 mg/mL concentration. The mixture was subjected to vortex mixing for 2 min to ensure homogeneity.

### Preparation of CUR-loaded liposomes

CUR-loaded liposomes were prepared using the thin-film hydration method. Briefly, soybean lecithin, cholesterol, and CUR were combined at a molar ratio of 6.15: 2.5: 0.6, respectively. The lipid and drug components were first dissolved in a chloroform solution. The organic solvent was then evaporated under reduced pressure using a rotary evaporator maintained at 55 °C and 100 rpm for 30 min, forming a thin lipid film on the inner wall of the flask. To ensure complete removal of any residual solvent, the film was further dried under vacuum for an additional 15 min. The resulting thin film was subsequently hydrated with 23 mL of distilled water under gentle agitation. The coarse multilamellar vesicle (MLV) dispersion obtained was then sonicated in a water bath sonicator for 2 min and briefly vortexed to yield a homogeneous suspension of CUR-Liposomes^12,13^.

### Fourier transform infrared spectroscopy (FTIR)

The molecular structure characterization of CUR-liposomes was analyzed by FTIR spectroscopy (JASCO 6100 spectrophotometer, Japan) at the Micro Analytical Center, Faculty of Science, Cairo University, Giza, Egypt.

1 mL of CUR-liposomes was centrifuged for 10 min at 12,000 rpm to extract the pellet for analysis. Spectra between 4000 and 400 cm^−1^ were obtained^14^.

### Evaluation of CUR-lipid association

The interaction between CUR and the liposomal components was evaluated by analyzing changes in their concentrations in the supernatant before and after centrifugation. The concentrations of both CUR and soy lecithin (lipid) in the supernatant were quantified using High-Performance Liquid Chromatography (HPLC Chromatograph YL- 9100 system) with a C-18 column (250 mm × 4.6 mm × 5 μm). The mobile phase consisted of acetonitrile and 5% acetic acid in a ratio of 75:25, delivered at a flow rate of 1 mL/min. Detection was performed at a wavelength of 425 nm.

### Dynamic light scattering (DLS)

The CUR-liposomes’ particle size and zeta potential were assessed using a DLS Zetasizer Nano series Nano ZS ZEN3600 (Malvern Instruments, UK) analyzer that yields data on molecular weight, concentration, zeta potential, and particle size^15^.

Before measurements were performed, the samples were diluted 100 times. Adjust the refractive index, temperature, and detecting angle to 25 °C and 90 °C, respectively. Select the appropriate cuvette for the sample and begin the analysis using a Helium-Neon Laser beam.

### Transmission electron microscopy (TEM)

Utilizing a JEM-2100 h TEM (JEOL, Japan; 200 kV) at the National Research Centre, Cairo, Egypt, the CUR liposomes’ shape and microstructure were examined. Before imaging, the sample was made by placing a drop of transmittance negative stain, phosphor tungsten acid, over a copper grid coated with carbon and letting it air dry.

### Differential scanning calorimetry (DSC)

The thermal behavior and phase transition temperature of the CUR-liposomes formulation were analyzed using DSC (Labsys evolution, SETARAM, France) at the National Research Center, Cairo, Egypt. The sample was placed in a sealed aluminum crucible and subjected to a heating scan from 30 °C to 80 °C under a controlled atmosphere. This analysis allowed for the determination of the phase transition temperature (Tm) and other relevant thermodynamic parameters of the lipid bilayer, providing insight into the stability and physical properties of the liposomal formulation.

### In vitro cell line study

A549, caco-2, PANC-1, PC3, MCF-7/ADR cells, and Vero cells from the Egyptian Holding Company for Biopharmaceuticals and Vaccines Production (Vacsera) in Cairo, Egypt, were used for the cytotoxicity test for both the CUR and CUR-liposomes samples. To create a full monolayer sheet, the cells were seeded at a density of about 1 × 10^5^ cells/mL (100 µl/well) and dispensed in a 96-well tissue culture plate. They were then incubated for 24 h at 37 °C. Following the formation of a confluent sheet of cells, the growth medium was decanted from 96-well microtiter plates, and the cell monolayer was twice washed with wash media. The tested material was diluted twice in RPMI medium (maintenance medium) containing 2% serum.

For each cell line, the following control groups were included: **Negative control**: untreated cells (maintenance medium only), **vehicle control**: cells treated with DMSO at the same final concentration present in the highest CUR treatment (300 µg/mL), and **positive control**: cells treated with doxorubicin (DOXO) at concentrations of 300, 100, 30, 10, 3, and 1 µg/mL. Each treatment concentration (including controls) was added at 0.1 mL per well. After being incubated at 37 °C, the plate was inspected. Physical indicators of toxicity, such as rounding, shrinkage, cell granulation, or partial or whole loss of the monolayer, were examined in the cells. A 5 mg/mL MTT solution in PBS was made (Bio Basic Canada Inc.). Each well received a 20 µL addition of MTT solution. To fully incorporate the MTT into the medium, place it on a shaking table and spin it at 150 rpm for five minutes. Allow the MTT to be metabolized by incubating it for four hours at 37 °C with 5% CO₂. Empty the media (if needed, dry the plate on paper towels to get rid of any residue).

Formazan, an MTT metabolic product, should be reconstituted in 200 µL of DMSO. To fully incorporate the formazan into the solvent, place it on a shaking table and spin it at 150 rpm for five minutes. At 560 nm, read the optical density; at 620 nm, subtract the background. There should be a direct relationship between optical density and cell number.

### Statistical analysis

Microsoft Excel’s Data Analysis ToolPak was used for statistical analysis. An unpaired t-test (assuming unequal variances) was performed to compare the cytotoxicity between free CUR and CUR-Liposomes in Vero cells, using the raw optical density (OD) values from three independent replicates (*n* = 3). The data are expressed as mean ± standard deviation (SD). Differences were considered statistically significant at *P* < 0.05.

## Results and discussion

### Molecular structure characterization

**Fig. 1:**
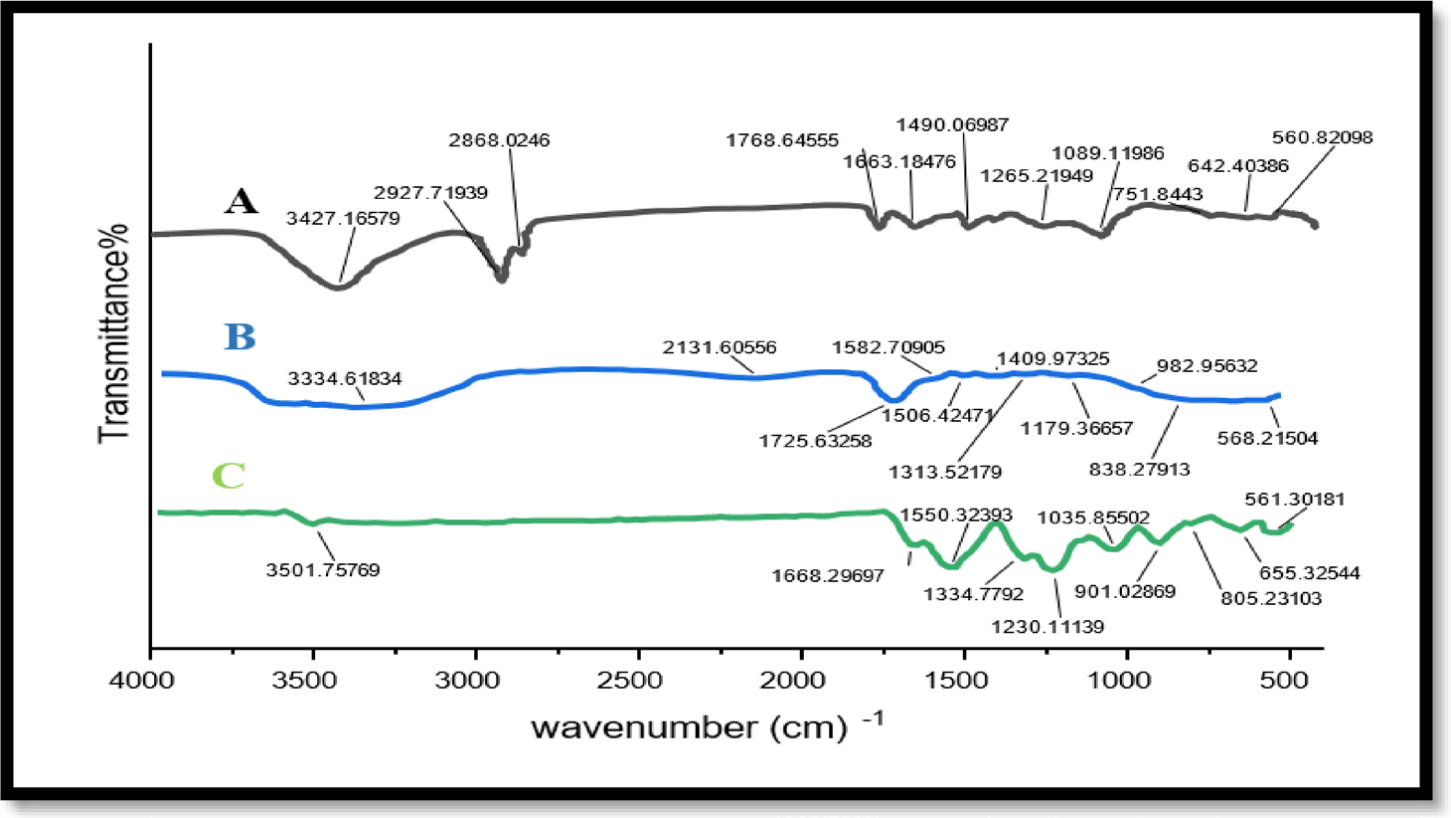
The FTIR spectra of CUR-liposomes (A), liposomes free (B), and CUR free(C).

The FTIR spectra of free CUR, free liposomes (soy lecithin and cholesterol), and CUR-Liposomes are shown in Fig. 1. The spectral analysis was conducted over the range of 4000–400 cm^−1^^16^.

#### CUR spectrum

CUR exhibited characteristic peaks at ~ 3510 cm^−1^ corresponding to O–H stretching^17^ (phenolic group), ~ 1627 cm^−1^ assigned to C = O and C = C stretching (conjugated diketone and aromatic groups), multiple bands in the range of 1270–1020 cm^−1^ attributed to C–O stretching and C–H bending. These peaks confirm the presence of hydroxyl, carbonyl, and aromatic functionalities in the CUR structure^18,19^.

#### Free liposomes spectrum

The liposomes without CUR showed a broad band at ~ 3400 cm^−1^ due to O–H stretching (from phospholipid and cholesterol), strong C–H stretching bands at ~ 2920 cm^−1^ and ~ 2850 cm^−1^, and a sharp ester C = O band at ~ 1735 cm^−1^^20,21^.

#### CUR-liposomes spectrum

The FTIR spectrum of CUR-liposomes exhibited a combination of peaks from both CUR and the liposomes structure. However, noticeable changes were observed: The O–H peak around ~ 3510 cm^−1^ became broader and less intense, indicating hydrogen bond formation between CUR and the lipid bilayer. The C = O and aromatic C = C peaks around ~ 1627 cm^−1^ were reduced and slightly shifted, suggesting interaction and possible encapsulation of CUR. The strong bands from the liposomes matrix, such as the C–H stretching (~ 2920 & 2850 cm^−1^) and ester C = O (~ 1735 cm^−1^), remained visible in CUR-Liposomes. The fingerprint region (~ 1230–1050 cm^−1^) also preserved the characteristic bands of phospholipids. These spectral changes confirm the successful encapsulation of CUR within the liposomal structure, with evidence of interaction between the active compound and the lipid components^13,21^.

### Evaluation of CUR-lipid association

The concentrations of both CUR and soy lecithin (lipid) in the supernatant were quantified using HPLC, the CUR-liposomes formulation was centrifuged at 5,000 rpm for 8 min to separate the liposomal pellet from the supernatant. The molar concentrations (µmol/mL) of both CUR and lipid in the supernatant, before and after centrifugation, were calculated using their respective molecular weights (CUR: 368.38 g/mol; soy lecithin: 643.97 g/mol). The change in lipid-to-CUR molar ratio was calculated as follows: Change in Molar Ratio = (Lipid/CUR molar ratio AFTER centrifugation) / (Lipid/CUR molar ratio BEFORE centrifugation). For our formulation, the lipid-to-CUR molar ratio in the supernatant decreased from 5.72 to 0.25 µmol/µmol after centrifugation, resulting in a change ratio of 0.04. This significant reduction indicates substantial co-precipitation of CUR with the liposomal lipid during the centrifugation process.

### Determination size and zeta potential

**Fig. 2:**
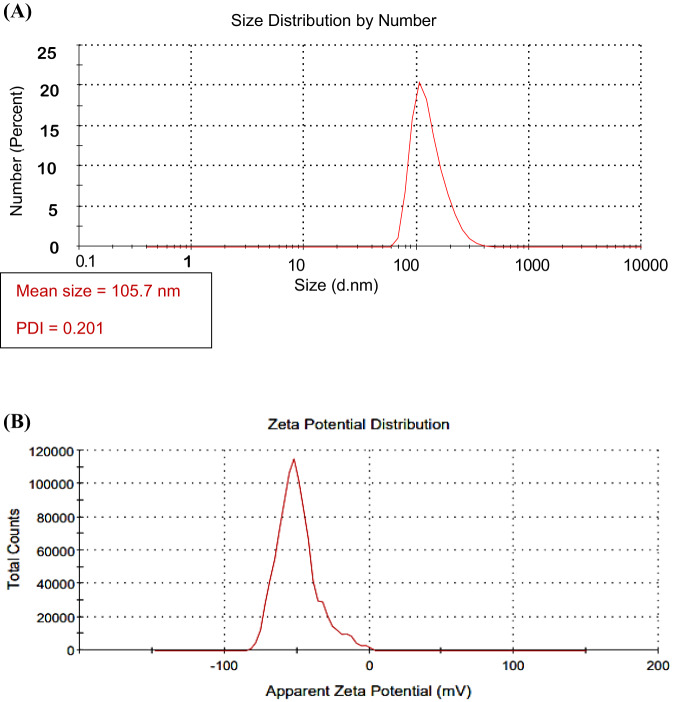
(**A**) DLS size distribution of CUR- loaded liposomes. (**B**) DLS image of the zeta potential of CUR-Liposomes.

The particle size and zeta potential of CUR-Liposomes were determined by a DLS Zetasizer Nano series Nano ZS ZEN3600 (Malvern Instruments, UK) analyzer, which provides information on particle size and zeta potential. DLS analysis revealed that the average hydrodynamic diameter of the CUR-loaded liposomes was 105.7 nm, as shown in Fig. 2a, with a polydispersity index (PDI) of 0.201, suggesting a relatively uniform size distribution. The single, well-defined peak suggests a monodisperse population of nanoparticles with minimal aggregation. The measured hydrodynamic diameter reflects the particle size in suspension, including the solvation layer, and is typically larger than the physical diameter observed under TEM, due to the contribution of the solvation layer and potential interparticle interactions in aqueous dispersion, while TEM measures the dry-state physical diameter of individual particles. These results confirm the nanoscale size and good homogeneity of the liposomal formulation.

The zeta potential of the CUR-loaded liposomes was measured to evaluate their colloidal stability. The results showed a high negative zeta potential value of -49.9 mV, with a major peak at -51.2 mV accounting for 95.5% of the total distribution, as shown in Figure 2b. This high magnitude of surface charge indicates strong electrostatic repulsion between particles, suggesting excellent stability of liposomal dispersion and a low tendency for aggregation over time. These values are within the optimal range for stable nanoparticle suspensions (±30 mV or more)^22,23^.

### Transmission electron microscopy (TEM)

**Fig. 3:**
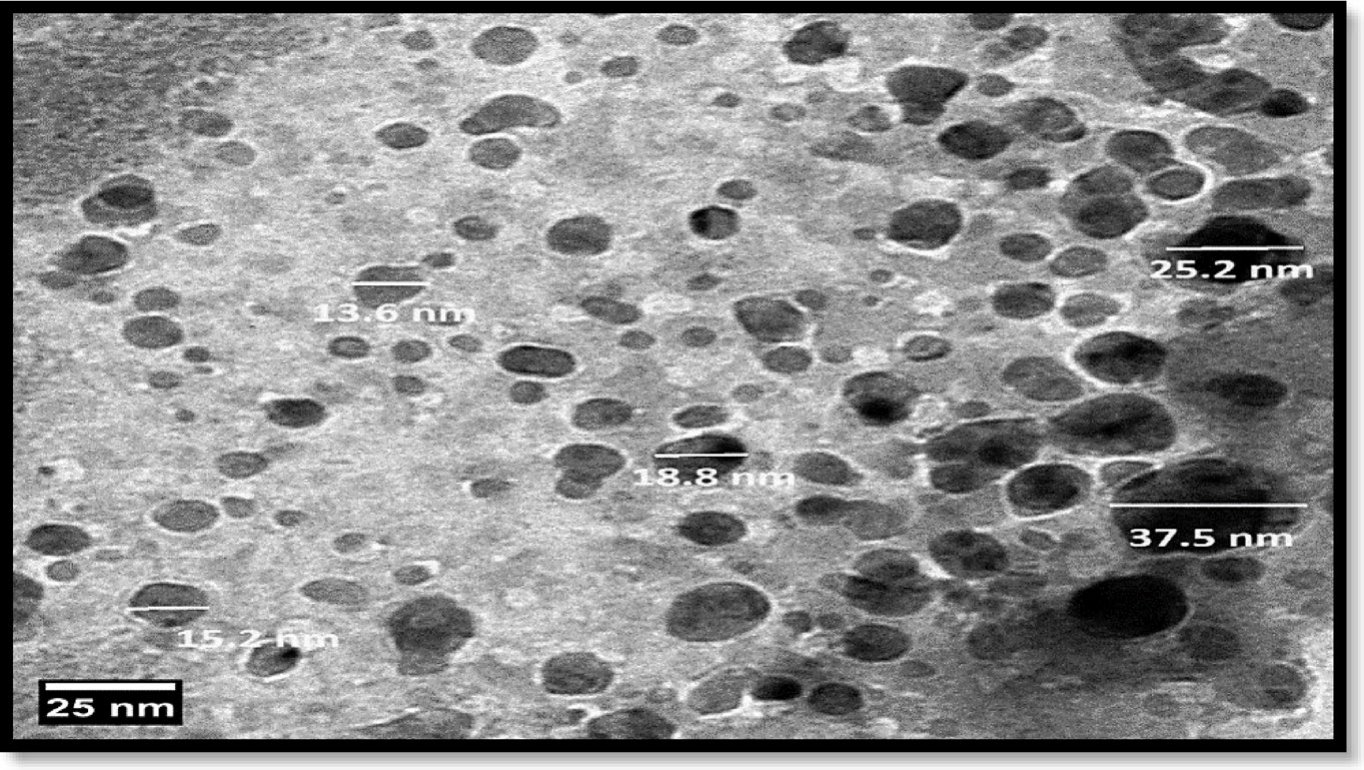
TEM image of CUR-Liposomes.

TEM provided additional confirmation of the sample’s detailed morphology. Any material structure change can be highlighted with this method. A drop from the liposomal dispersion was applied onto carbon-coated copper that was set on filter paper to create the sample. Before imaging, let it air dry for a few seconds, then apply a drop of transmittance-negative stain. As illustrated in Fig. 3, because of CUR, the TEM pictures showed black patches inside the liposomes, which suggested the existence of electron-dense components. Its breadth ranges from 13.6 nm to 37.5 nm, and it likewise had a multilamellar appearance, demonstrating the creation of concentric lipid bilayers within the liposomes. TEM scans demonstrated that the size range of CUR-Liposomes was within the range of their therapeutic potential^24^.

### Differential scanning calorimetry (DSC)

**Fig. 4:**
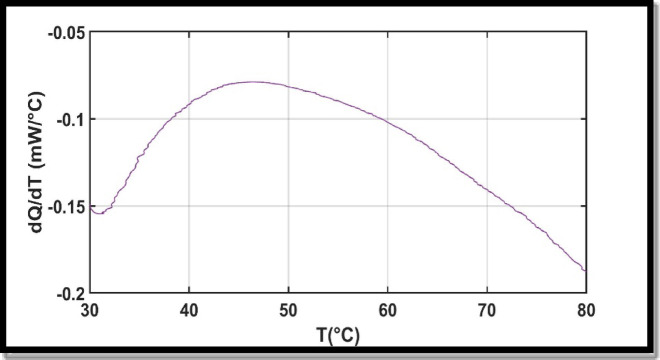
DSC curve for CUR-loaded.

DSC analysis has been performed on our sample to study the effect of temperature on the synthesized liposomes, mainly to determine the phase transition temperature. In Fig. 4, the thermogram revealed a broad endothermic transition around 45 °C, which corresponds to the main phase transition temperature (Tm) of the lipid bilayer. This transition reflects the change from a gel phase (ordered structure) to a liquid crystalline phase (disordered structure), indicating increased fluidity of the liposomal membrane. This influential parameter reflects the liposomes permeability toward the drug by describing the membrane fluidity.

### Cell line cytotoxicity

**Fig. 5:**
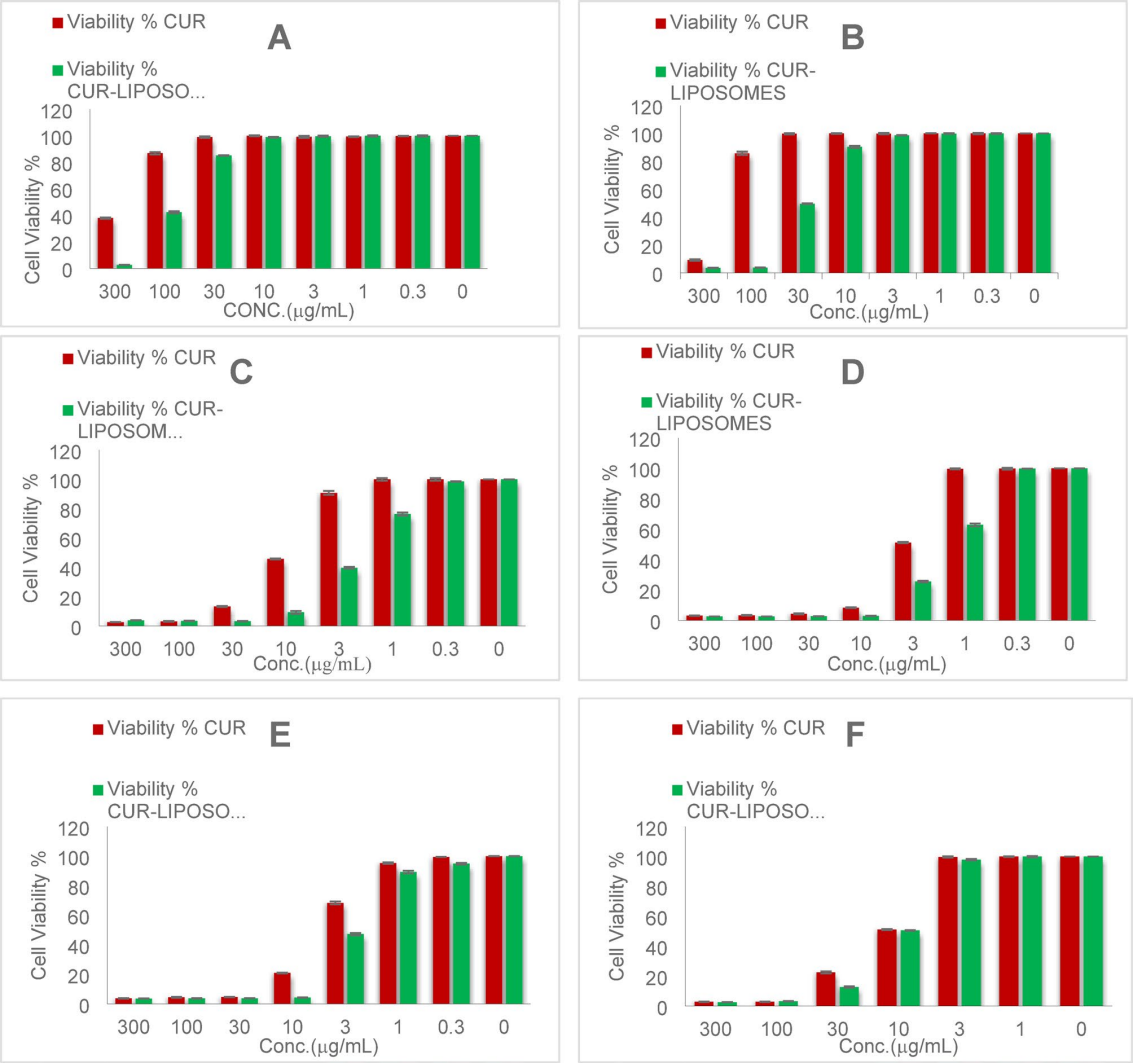
Cell viability of (**A**) MCF-7/ADR cells, (**B**) A549 cells, (**C**) Caco-2 cells, (**D**) PANC-1 cells, (**E**) PC-3 cells, and (**F**) Vero (normal) cells after exposure to 300, 100, 30, 10, 3, 1, and 0.3 µg/mL of CUR and CUR-Liposomes. Red bars represent CUR and green bars represent CUR-Liposomes, the values are expressed as mean ± SEM (*n* = 3).

The biocompatibility of synthesized CUR-liposomes as drug delivery systems in MCF-7/ADR cells (Table 1), A549 (Table 2), caco-2(Table 3), PANC-1(Table 4), PC3 (Table 5), and Vero cells (Table 6) in both different concentrations of CUR and CUR-liposomes was assessed using cytotoxicity assays. The histograms of all cell viability analyses are presented in Fig. 5. The cell viability assay, as demonstrated in Table 1; Fig. 5a, is effective in assessing the impact of drug candidates on cells and can help refine cell culture conditions. The results indicate that CUR-Liposomes impedes cell growth. The Mcf7ADR cell line was treated with varying concentrations of CUR and CUR-Liposomes (300, 100, 30, 10, 3, 1, and 0.3 µg/mL) separately. A comparison of cell viability across all concentrations of CUR-Liposomes with that of CUR shows a notable difference, with a decline in cell viability from lower to higher concentrations in CUR-Liposomes, while cell viability remains relatively stable at lower concentrations for CUR. The IC50 ± SD values for CUR and CUR-Liposomes were 249.85 ± 1.8 and 87.43 ± 1.18, respectively.

As presented in Table 2; Fig. 5b, the A549 cell line was also subjected to 300, 100, 30, 10, 3, 1, and 0.3 µg/mL of both CUR and CUR-Liposomes separately. The IC50 ± SD for CUR and CUR-Liposomes were 185.8 ± 0.95 and 44.43 ± 0.38. In Table 3; Fig. 5c, the Caco2 cell line was treated with the same concentrations of CUR and CUR-Liposomes separately. The IC50 ± SD results were 9.34 ± 0.04 for CUR and 2.44 ± 0.01 for CUR-Liposomes. In Table 4; Fig. 5d, the PANC1 cell line underwent exposure to CUR and CUR-Liposomes at the same concentrations. The IC50 ± SD values were 4.96 ± 0.02 for CUR and 1.89 ± 0.02 for CUR-Liposomes. In Table 5; Fig. 5e, the (Pc3) cell line was exposed to CUR and CUR-Liposomes at the same concentrations. The IC50 ± SD of both CUR and CUR-Liposomes was 6.1 ± 0.09 and 2.88 ± 0.05. For the normal Vero cell line, which was exposed to the same concentrations of CUR and CUR-Liposomes (as shown in Table 6; Fig. 5f), the IC50 ± SD was 16.9 ± 0.2 for CUR and 15.49 ± 0.05 for CUR-Liposomes.

The IC50 values obtained from cytotoxicity assays indicated that CUR-Liposomes was notably more effective than free CUR across all investigated cancer cell lines (MCF-7/ADR, A549, Caco-2, PANC-1, and PC3). In every instance, a lower concentration of CUR-Liposomes was necessary to achieve 50% inhibition of cell viability in comparison to free CUR, emphasizing the increased anticancer effectiveness of the liposomal formulation. Thus, it can be inferred that CUR-Liposomes formulations are biocompatible, and suitable for drug delivery systems^25^. Significantly, in Vero cells (a normal cell line), the IC50 values for CUR and CUR-Liposomes were quite similar (16.9 µg/mL and 15.49 µg/mL, respectively). Statistical analysis (unpaired t-test, *p* > 0.05) confirmed that this difference was not significant, indicating that liposomal encapsulation did not enhance toxicity toward normal cells. This trend indicates that although CUR-Liposomes boost cytotoxic effects in cancer cells, it retains a relatively safe profile in non-cancerous cells.

In summary, these findings reveal that the liposomal encapsulation of CUR enhances its anticancer effectiveness, enabling significant cytotoxicity at lower concentrations than free CUR, while not markedly increasing toxicity in normal cells^26^. The inclusion of the vehicle control (DMSO) confirmed that the solvent itself had no observable cytotoxic effect at the final concentration used. Additionally, doxorubicin, used as a positive control, produced the expected marked reduction in cell viability, confirming the responsiveness of the assay.

**Fig. 6:**
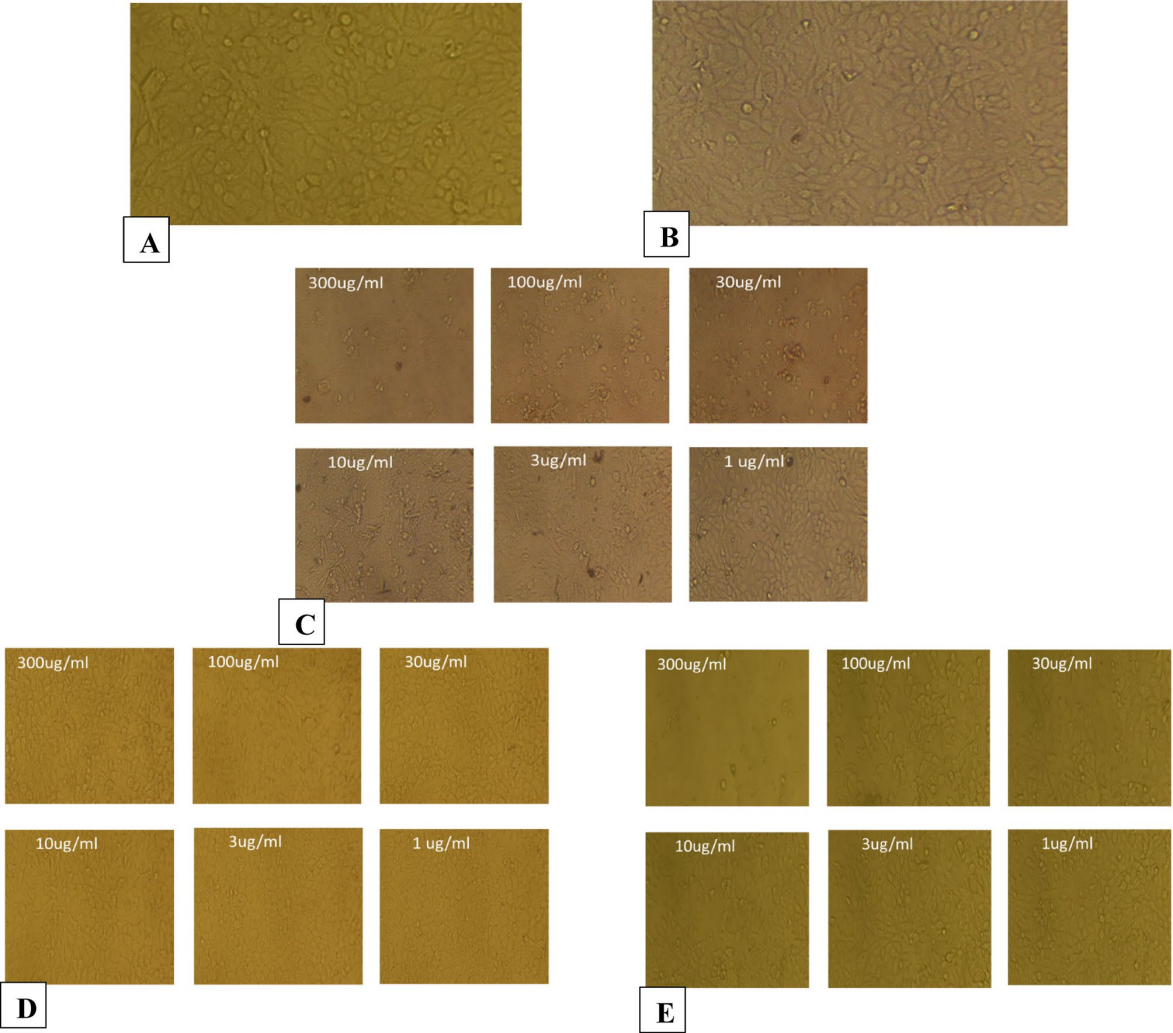
Representative morphology of MCF-7/ADR cells. (**A**) Untreated cells (Negative Control). (**B**) Cells treated with DMSO (Vehicle Control; same final DMSO concentration used in the 300 µg/mL treatment). (**C**) Cells treated with DOXO at 300, 100, 30, 10, 3, and 1 µg/mL, respectively (Positive Control). (**D**) Cells treated with free CUR at 300, 100, 30, 10, 3, and 1 µg/mL, respectively. (**E**) Cells treated with CUR-Liposomes at 300, 100, 30, 10, 3, and 1 µg/mL, respectively.

**Fig. 7:**
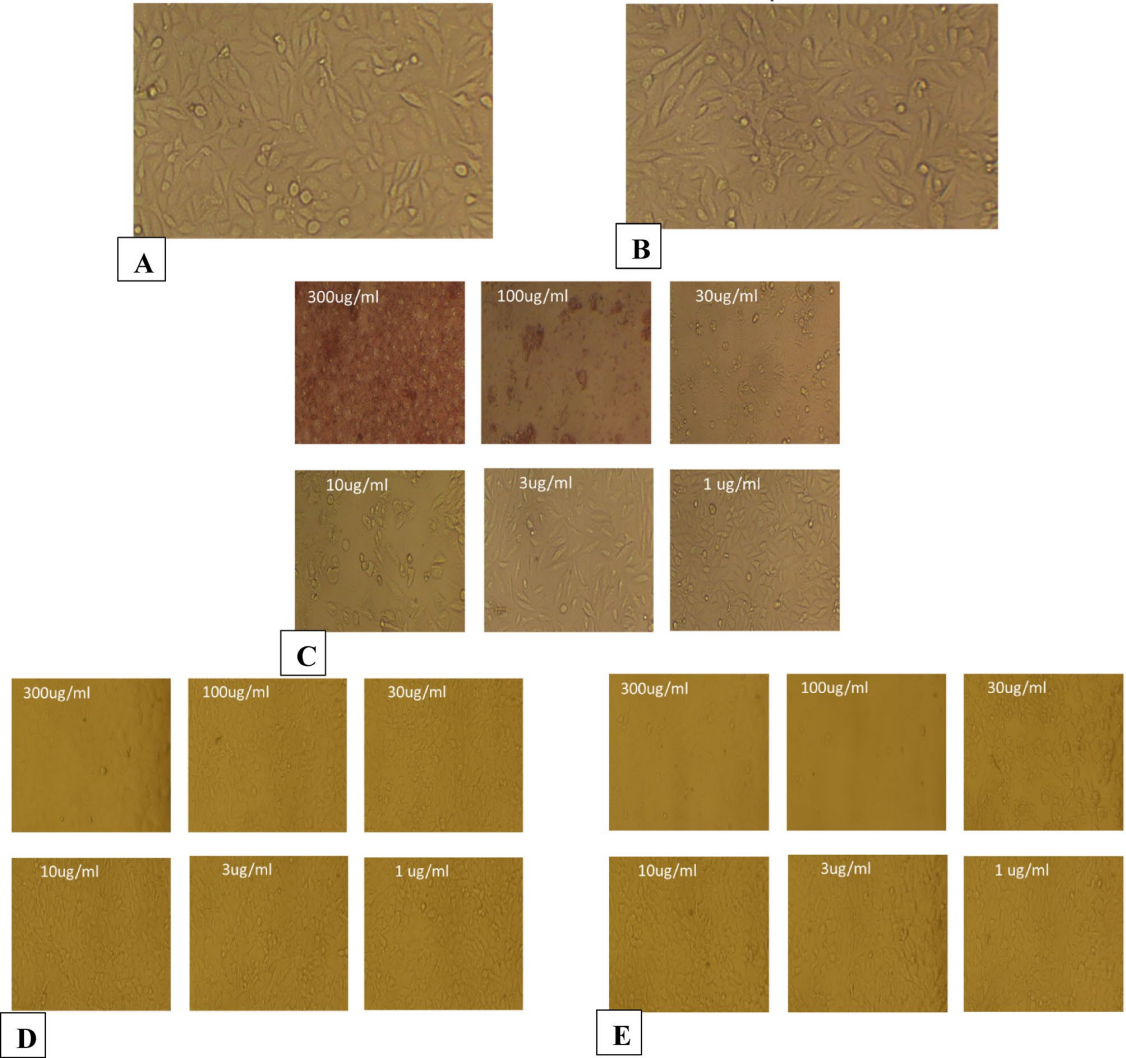
Representative morphology of A549 cells. (**A**) Untreated cells (Negative Control). (**B**) Cells treated with DMSO (Vehicle Control; same final DMSO concentration used in the 300 µg/mL treatment). (**C**) Cells treated with DOXO at 300, 100, 30, 10, 3, and 1 µg/mL, respectively (Positive Control). (**D**) Cells treated with free CUR at 300, 100, 30, 10, 3, and 1 µg/mL, respectively. (**E**) Cells treated with CUR-Liposomes at 300, 100, 30, 10, 3, and 1 µg/mL, respectively.

**Fig. 8:**
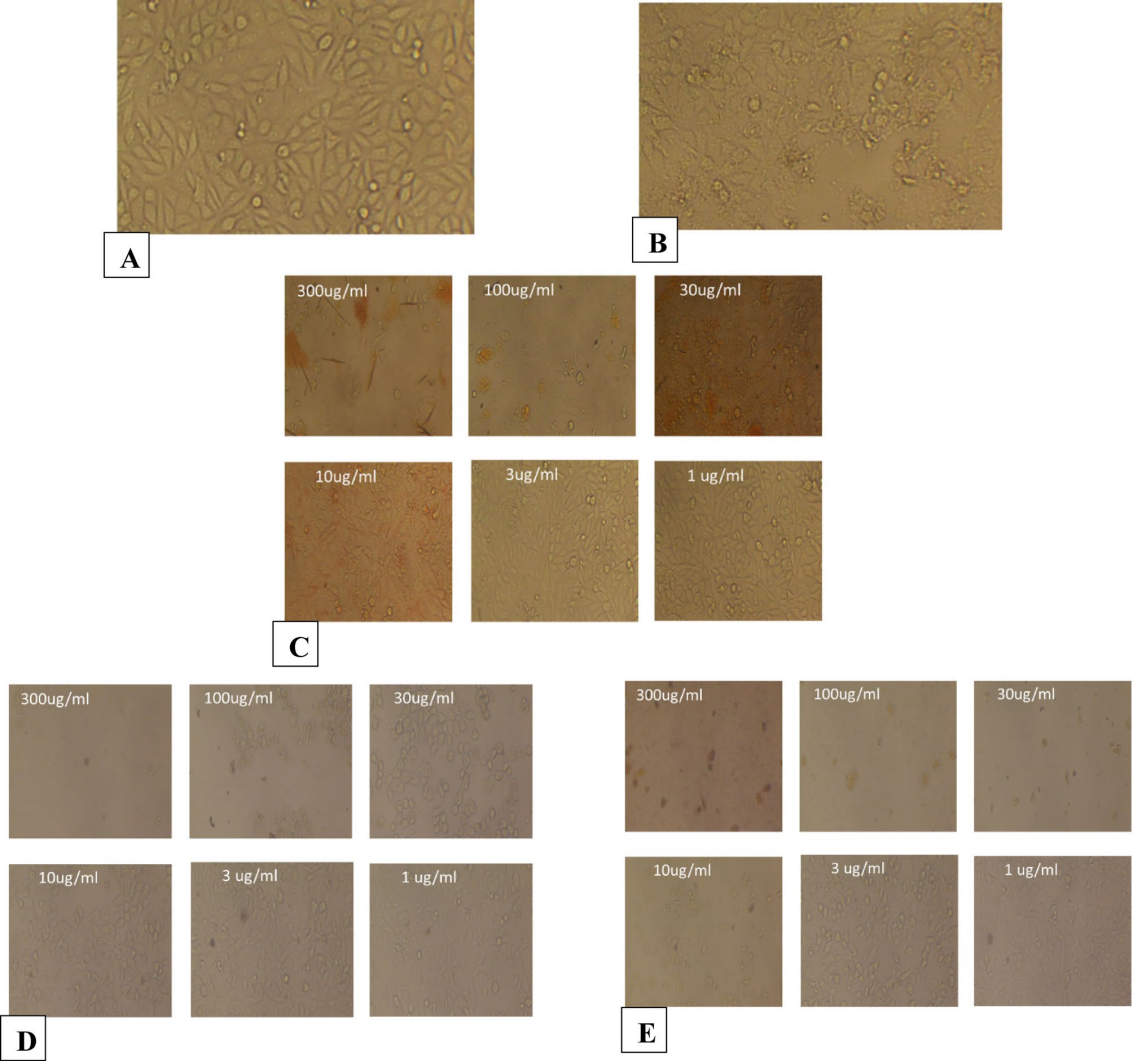
Representative morphology of Caco2 cells. (**A**) Untreated cells (Negative Control). (**B**) Cells treated with DMSO (Vehicle Control; same final DMSO concentration used in the 300 µg/mL treatment). (**C**) Cells treated with DOXO at 300, 100, 30, 10, 3, and 1 µg/mL, respectively (Positive Control). (**D**) Cells treated with free CUR at 300, 100, 30, 10, 3, and 1 µg/mL, respectively. (**E**) Cells treated with CUR-Liposomes at 300, 100, 30, 10, 3, and 1 µg/mL, respectively.

**Fig. 9:**
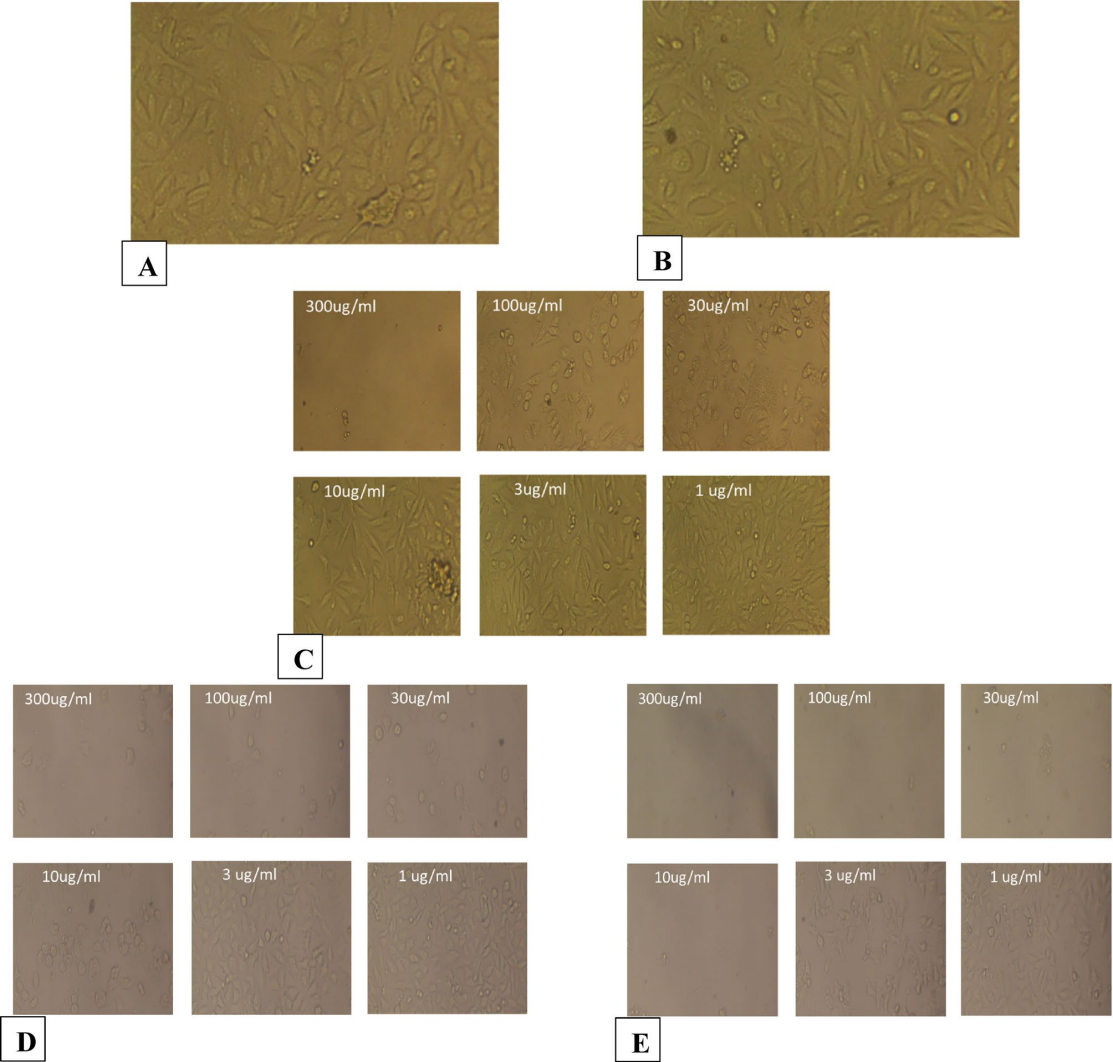
Representative morphology of PANC1 cells. (**A**) Untreated cells (Negative Control). (**B**) Cells treated with DMSO (Vehicle Control; same final DMSO concentration used in the 300 µg/mL treatment). (**C**) Cells treated with DOXO at 300, 100, 30, 10, 3, and 1 µg/mL, respectively (Positive Control). (**D**) Cells treated with free CUR at 300, 100, 30, 10, 3, and 1 µg/mL, respectively. (**E**) Cells treated with CUR-Liposomes at 300, 100, 30, 10, 3, and 1 µg/mL, respectively.

**Fig. 10:**
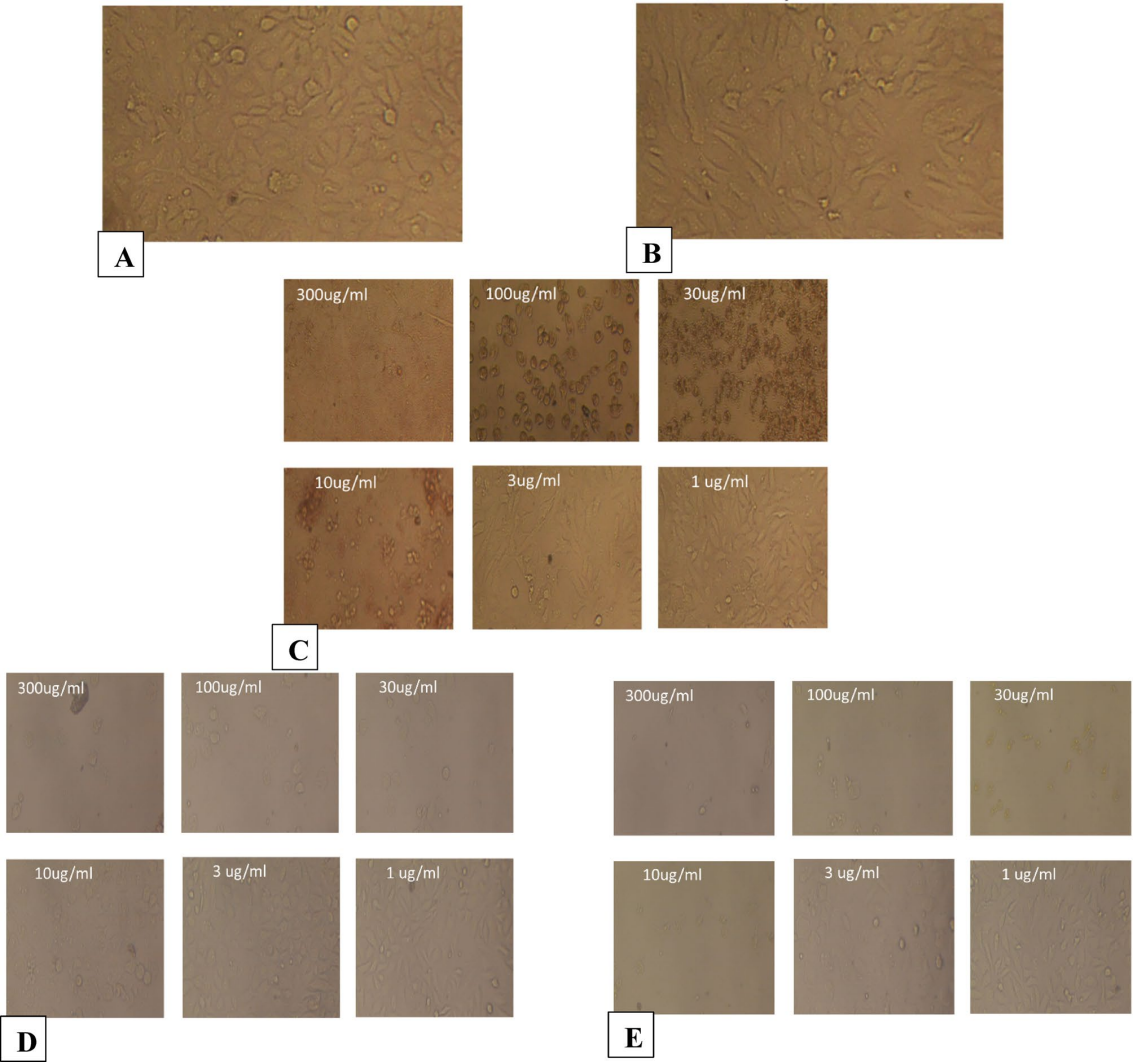
Representative morphology of PC3 cells. (**A**) Untreated cells (Negative Control). (**B**) Cells treated with DMSO (Vehicle Control; same final DMSO concentration used in the 300 µg/mL treatment). (**C**) Cells treated with DOXO at 300, 100, 30, 10, 3, and 1 µg/mL, respectively (Positive Control). (**D**) Cells treated with free CUR at 300, 100, 30, 10, 3, and 1 µg/mL, respectively. (**E**) Cells treated with CUR-Liposomes at 300, 100, 30, 10, 3, and 1 µg/mL, respectively.

**Fig. 11:**
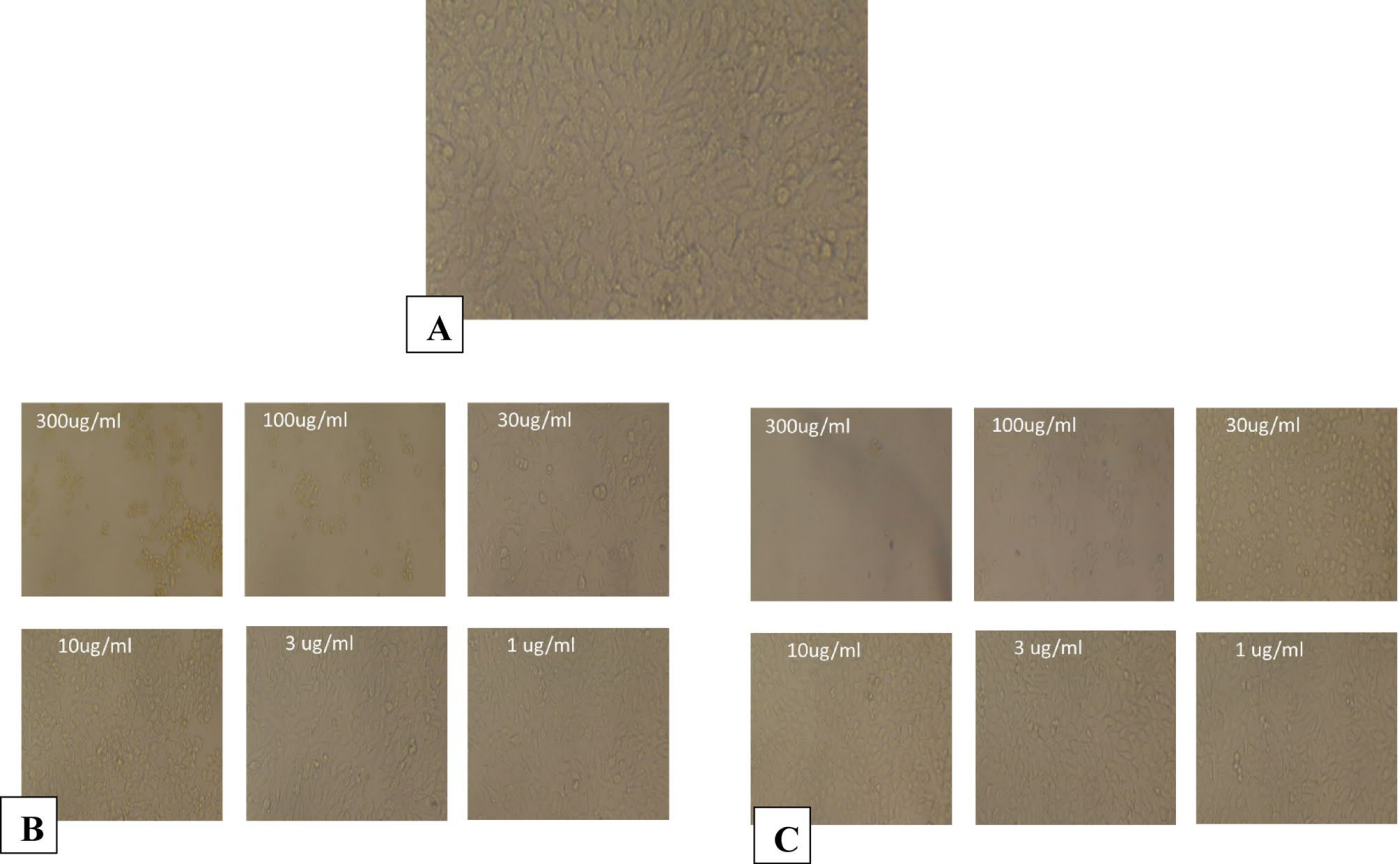
Representative morphology of Vero cells. (**A**) Control: untreated cells. (**B**) Cells treated with free CUR at 300, 100, 30, 10, 3, and 1 µg/mL. (**C**) Cells treated with CUR-Liposomes at 300, 100, 30, 10, 3, and 1 µg/mL.

**Fig. 12:**
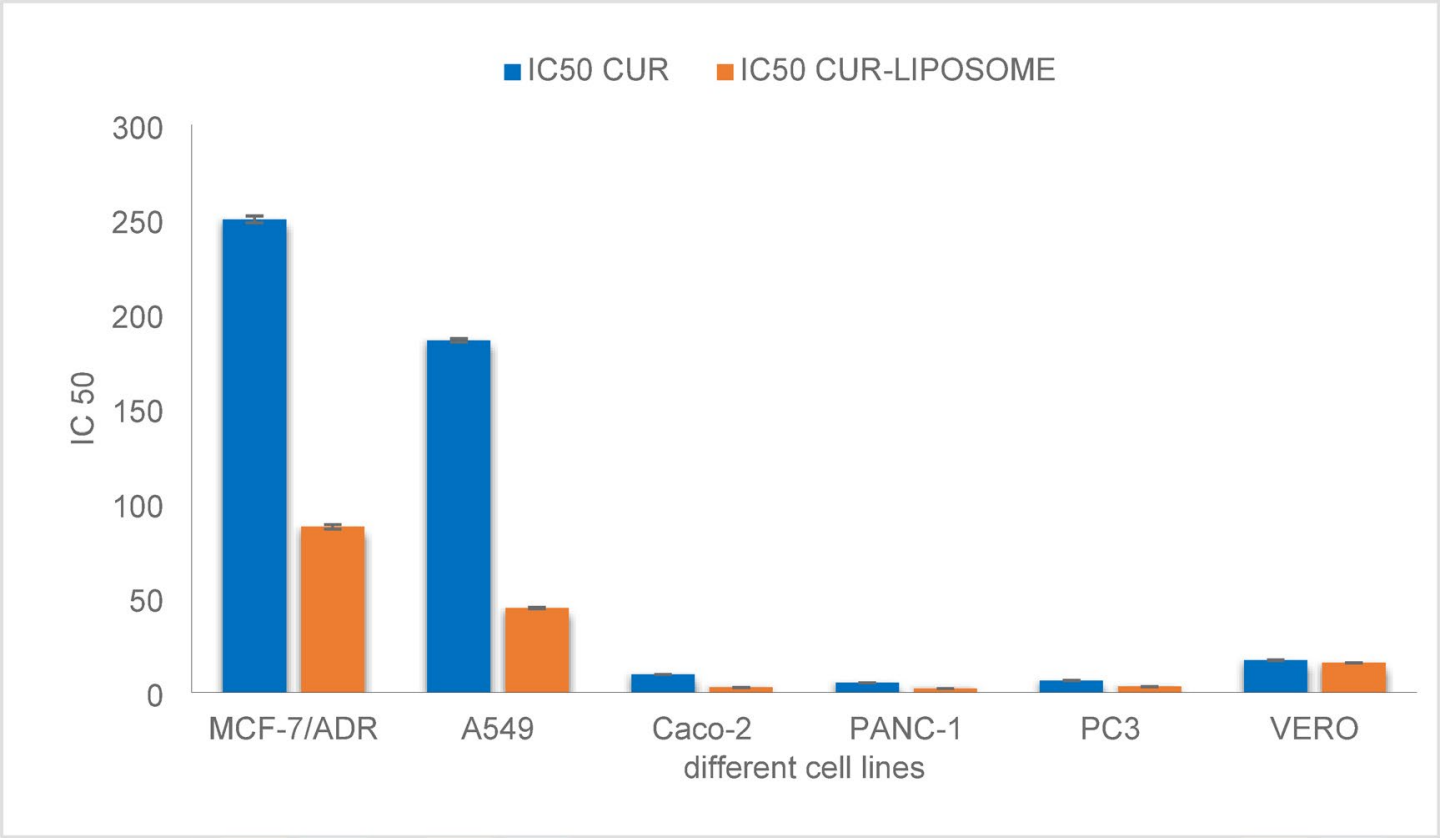
The graph represents the cytotoxicity of CUR and CUR-Liposomes on different cell lines as indicated by their IC50 value. blue bars represent CUR, and orange bars represent CUR-Liposomes. The values are expressed as mean ± SD.

The morphological observations are shown in Figs. 6, 7, 8, 9, 10 and 11, where each panel includes: untreated cells (negative control), DMSO-treated cells (vehicle control), doxorubicin-treated cells (positive control), free CUR-treated cells, and CUR-Liposomes-treated cells. Clear differences were observed between free CUR and CUR-Liposomes, particularly at higher concentrations, where CUR-Liposomes appeared to induce more pronounced morphological changes in cancer cells. Cytotoxicity, as represented by the IC50 values for free CUR and CUR-Liposomes across different cell lines, is evident in Fig. 12. While CUR-Liposomes generally exhibited lower IC50 values than free CUR in cancer cell lines, indicating enhanced cytotoxic potency, both formulations showed comparable effects in Vero cells. These findings suggest that liposomal encapsulation improves CUR cytotoxicity toward cancer cells without substantially increasing toxicity in normal cells.

**Table 1:** Assessment of chemosensitivity in (MCF-7/ADR) cell lines utilizing the MTT assay through half-log concentration dilutions. This table indicates that the results were represented as IC50, which refers to the concentration of the cytotoxic drug that decreases cell viability by 50% compared to the control.

ID	µg/mL	O.D			Mean O.D	±SEM	Viability %	Toxicity %	IC50 ± SD
MCF-7/ADR	–	0.674	0.669	0.666	0.669667	0.0402	100	0	µg/mL
DMSO	300	0.723	0.728	0.719	0.723333	0.0026	98.01264679	1.987353207	
Doxo.	300	0.02	0.018	0.021	0.019667	0.0009	2.664859982	97.33514002	5.04 ± 0.09
	100	0.022	0.019	0.024	0.021667	0.0015	2.935862692	97.06413731	
	30	0.022	0.022	0.025	0.023	0.0010	3.116531165	96.88346883	
	10	0.087	0.094	0.081	0.087333	0.0038	11.833785	88.166215	
	3	0.362	0.377	0.359	0.366	0.0056	49.59349593	50.40650407	
	1	0.736	0.739	0.733	0.736	0.0017	99.72899729	0.27100271	
	0.31	0.734	0.739	0.736	0.736333	0.0015	99.77416441	0.225835592	
CUR	300	0.26	0.248	0.255	0.254333	0.0035	37.97907517	62.02092483	249.85 ± 1.8
	100	0.573	0.589	0.583	0.581667	0.0047	86.85909066	13.14090934	
	30	0.664	0.658	0.668	0.663333	0.0029	99.05420654	0.945793457	
	10	0.67	0.674	0.665	0.669667	0.0026	99.99995022	4.9776E-05	
	3	0.658	0.668	0.666	0.664	0.0031	99.15375851	0.84624149	
	1	0.667	0.668	0.661	0.665333	0.0022	99.35286244	0.647137557	
	0.3	0.671	0.668	0.667	0.668667	0.0012	99.85062227	0.149377726	
CUR-Liposomes	300	0.019	0.017	0.02	0.018667	0.0009	2.578268877	97.42173112	87.43 ± 1.18
	100	0.316	0.297	0.306	0.306333	0.0055	42.31123389	57.68876611	
	30	0.62	0.618	0.611	0.616333	0.0027	85.12891344	14.87108656	
	10	0.718	0.72	0.712	0.716667	0.0024	98.98710866	1.012891344	
	3	0.722	0.721	0.723	0.722	0.0006	99.72375691	0.276243094	
	1	0.725	0.72	0.726	0.723667	0.0019	99.95395948	0.046040516	
	0.3	0.722	0.724	0.725	0.723667	0.0009	99.95395948	0.046040516	

**Table 2:** Assessment of chemosensitivity in (A549) cell lines utilizing the MTT assay through half-log concentration dilutions. This table indicates that the results were represented as IC50, which refers to the concentration of the cytotoxic drug that decreases cell viability by 50% compared to the control.

ID	µg/mL	O.D			Mean O.D	±SEM	Viability %	Toxicity %	IC50± SD
A549	–	0.717	0.72	0.714	0.717	0.0017	100	0	µg/mL
DMSO	300	0.636	0.622	0.627	0.628333	0.0041	93.36305102	6.636948985	
Doxo.	300	0.02	0.02	0.02	0.02	0	2.971768202	97.0282318	2.74 ± 0.03
	100	0.02	0.02	0.02	0.02	0	2.971768202	97.0282318	
	30	0.026	0.023	0.028	0.025667	0.0015	3.813769193	96.18623081	
	10	0.108	0.115	0.111	0.111333	0.0020	16.54284299	83.45715701	
	3	0.287	0.293	0.302	0.294	0.0044	43.68499257	56.31500743	
	1	0.669	0.674	0.673	0.672	0.0015	99.85141159	0.14858841	
	0.31	0.672	0.67	0.674	0.672	0.0012	99.85141159	0.14858841	
CUR	300	0.059	0.073	0.066	0.066	0.0040	9.205020921	90.79497908	185.8 ± 0.95
	100	0.625	0.6	0.618	0.614333	0.0074	85.68107857	14.31892143	
	30	0.715	0.711	0.718	0.714667	0.0020	99.67456997	0.325430033	
	10	0.719	0.716	0.714	0.716333	0.0015	99.90701999	0.092980009	
	3	0.718	0.713	0.717	0.716	0.0015	99.86052999	0.139470014	
	1	0.718	0.719	0.714	0.717	0.0015	100	0	
	0.3	0.72	0.713	0.716	0.716333	0.0020	99.90701999	0.092980009	
CUR-Liposomes	300	0.024	0.025	0.028	0.025667	0.0012	3.579730358	96.42026964	44.43 ± 0.38
	100	0.027	0.024	0.029	0.026667	0.0015	3.719200372	96.28079963	
	30	0.354	0.36	0.351	0.355	0.0026	49.51185495	50.48814505	
	10	0.658	0.642	0.644	0.648	0.0050	90.37656904	9.623430962	
	3	0.708	0.711	0.703	0.707333	0.0023	98.65178987	1.348210135	
	1	0.719	0.714	0.715	0.716	0.0015	99.86052999	0.139470014	
	0.3	0.713	0.722	0.715	0.716667	0.0027	99.95351	0.046490005	

**Table 3:** Assessment of chemosensitivity in (Caco2) cell lines utilizing the MTT assay through half-log concentration dilutions. This table indicates that the results were represented as IC50, which refers to the concentration of the cytotoxic drug that decreases cell viability by 50% compared to the control.

ID	µg/mL	O.D			Mean O.D	±SEM	Viability %	Toxicity %	IC50 ± SD
Caco2	–	0.66	0.67	0.674	0.668	0.0042	100	0	µg/mL
DMSO	300	0.525	0.518	0.506	0.516333	0.0055	72.0130172	27.9869828	
Doxo.	300	0.02	0.02	0.02	0.02	0	2.789400279	97.21059972	16.49 ± 0.09
	100	0.02	0.023	0.021	0.021333	0.0009	2.975360298	97.0246397	
	30	0.146	0.151	0.138	0.145	0.0038	20.22315202	79.77684798	
	10	0.355	0.359	0.367	0.360333	0.0035	50.25569503	49.74430497	
	3	0.721	0.71	0.716	0.715667	0.0032	99.81403998	0.185960019	
	1	0.718	0.714	0.719	0.717	0.0015	100	0	
	0.31	0.72	0.714	0.717	0.717	0.0017	100	0	
CUR	300	0.018	0.018	0.017	0.017667	0.0003	2.644710579	97.35528942	9.34 ± 0.04
	100	0.022	0.019	0.023	0.021333	0.0012	3.193612774	96.80638723	
	30	0.086	0.093	0.087	0.088667	0.0022	13.27345309	86.72654691	
	10	0.298	0.31	0.307	0.305	0.0036	45.65868263	54.34131737	
	3	0.616	0.597	0.606	0.606333	0.0055	90.76846307	9.231536926	
	1	0.671	0.664	0.668	0.667667	0.0020	99.9500998	0.0499002	
	0.3	0.67	0.669	0.665	0.668	0.0015	100	0	
CUR-Liposomes	300	0.026	0.025	0.027	0.026	0.0006	3.892215569	96.10778443	2.44 ± 0.01
	100	0.021	0.025	0.024	0.023333	0.0012	3.493013972	96.50698603	
	30	0.02	0.024	0.023	0.022333	0.0012	3.343313373	96.65668663	
	10	0.074	0.055	0.058	0.062333	0.0059	9.331337325	90.66866267	
	3	0.254	0.269	0.273	0.265333	0.0058	39.72055888	60.27944112	
	1	0.517	0.505	0.508	0.51	0.0036	76.34730539	23.65269461	
	0.3	0.653	0.659	0.663	0.658333	0.0029	98.55289421	1.447105788	

**Table 4:** Assessment of chemosensitivity in (PANC1) cell lines utilizing the MTT assay through half-log concentration dilutions. This table indicates that the results were represented as IC50, which refers to the concentration of the cytotoxic drug that decreases cell viability by 50% compared to the control.

ID	µg/mL	O.D			Mean O.D	±SEM	Viability %	Toxicity %	IC50 ± SD
PANC1	–	0.688	0.691	0.697	0.692	0.0026	100	0	µg/mL
DMSO	300	0.648	0.641	0.637	0.642	0.0032	98.16513761	1.834862385	
Doxo.	300	0.018	0.018	0.019	0.018333	0.0003	2.803261978	97.19673802	8.41 ± 0.05
	100	0.026	0.023	0.026	0.025	0.0010	3.822629969	96.17737003	
	30	0.095	0.083	0.104	0.094	0.0061	14.37308869	85.62691131	
	10	0.241	0.246	0.25	0.245667	0.0026	37.5637105	62.4362895	
	3	0.65	0.638	0.647	0.645	0.0036	98.62385321	1.376146789	
	1	0.659	0.657	0.655	0.657	0.0012	100.4587156	0	
	0.31	0.657	0.65	0.655	0.654	0.0021	100	0	
CUR	300	0.023	0.022	0.02	0.021667	0.0009	3.131021195	96.86897881	4.96 ± 0.02
	100	0.021	0.026	0.022	0.023	0.0015	3.323699422	96.67630058	
	30	0.027	0.033	0.029	0.029667	0.0018	4.287090559	95.71290944	
	10	0.054	0.06	0.058	0.057333	0.0018	8.285163776	91.71483622	
	3	0.354	0.357	0.351	0.354	0.0017	51.15606936	48.84393064	
	1	0.69	0.686	0.693	0.689667	0.0020	99.6628131	0.337186898	
	0.3	0.693	0.687	0.69	0.69	0.0017	99.71098266	0.289017341	
CUR-Liposomes	300	0.018	0.017	0.018	0.017667	0.0003	2.552986513	97.44701349	1.89 ± 0.02
	100	0.018	0.018	0.018	0.018	0	2.601156069	97.39884393	
	30	0.018	0.019	0.018	0.018333	0.0003	2.649325626	97.35067437	
	10	0.019	0.022	0.018	0.019667	0.0012	2.842003854	97.15799615	
	3	0.169	0.178	0.183	0.176667	0.0041	25.52986513	74.47013487	
	1	0.438	0.421	0.444	0.434333	0.0069	62.76493256	37.23506744	
	0.3	0.687	0.689	0.695	0.690333	0.0024	99.75915222	0.240847784	

**Table 5:** Assessment of chemosensitivity in (PC3) cell lines utilizing the MTT assay through half-log concentration dilutions. This table indicates that the results were represented as IC50, which refers to the concentration of the cytotoxic drug that decreases cell viability by 50% compared to the control.

ID	µg/mL	O.D			Mean O.D	±SEM	Viability %	Toxicity %	IC50 ± SD
PC3	–	0.529	0.532	0.535	0.532	0.0017	100	0	µg/mL
DMSO	300	0.538	0.52	0.526	0.528	0.0053	88.29431438	11.70568562	
Doxo.	300	0.02	0.02	0.02	0.02	0	3.344481605	96.65551839	2.23 ± 0.06
	100	0.02	0.02	0.02	0.02	0	3.344481605	96.65551839	
	30	0.02	0.02	0.02	0.02	0	3.344481605	96.65551839	
	10	0.024	0.023	0.027	0.024667	0.0012	4.124860647	95.87513935	
	3	0.186	0.199	0.175	0.186667	0.0069	31.21516165	68.78483835	
	1	0.473	0.48	0.467	0.473333	0.0038	79.15273133	20.84726867	
	0.31	0.593	0.597	0.595	0.595	0.0012	99.49832776	0.501672241	
CUR	300	0.02	0.019	0.022	0.020333	0.0009	3.822055138	96.17794486	6.1 ± 0.09
	100	0.024	0.021	0.026	0.023667	0.0015	4.448621554	95.55137845	
	30	0.024	0.024	0.027	0.025	0.0010	4.69924812	95.30075188	
	10	0.111	0.109	0.114	0.111333	0.0015	20.9273183	79.0726817	
	3	0.37	0.354	0.367	0.363667	0.0049	68.35839599	31.64160401	
	1	0.51	0.504	0.507	0.507	0.0017	95.30075188	4.69924812	
	0.3	0.526	0.53	0.531	0.529	0.0015	99.43609023	0.563909774	
CUR-Liposomes	300	0.02	0.019	0.019	0.019333	0.0003	3.634085213	96.36591479	2.88 ± 0.05
	100	0.019	0.022	0.019	0.02	0.0010	3.759398496	96.2406015	
	30	0.02	0.02	0.021	0.020333	0.0003	3.822055138	96.17794486	
	10	0.023	0.021	0.025	0.023	0.0012	4.323308271	95.67669173	
	3	0.243	0.258	0.253	0.251333	0.0044	47.24310777	52.75689223	
	1	0.463	0.477	0.485	0.475	0.0064	89.28571429	10.71428571	
	0.3	0.502	0.499	0.513	0.504667	0.0043	94.86215539	5.137844612	

**Table 6:** Assessment of chemosensitivity in Vero cell lines utilizing the MTT assay through half-log concentration dilutions. This table indicates that the results were represented as IC50, which refers to the concentration of the cytotoxic drug that decreases cell viability by 50% compared to the control.

ID	µg/mL	O.D			Mean O.D	±SEM	Viability %	Toxicity %	IC50 ± SD
Vero	–	0.726	0.724	0.731	0.727	0.0021	100	0	µg/mL
CUR	300	0.023	0.02	0.021	0.021333	0.0009	2.934433746	97.06556625	16.9 ± 0.2
	100	0.02	0.02	0.024	0.021333	0.0013	2.934433746	97.06556625	
	30	0.165	0.172	0.155	0.164	0.0049	22.55845942	77.44154058	
	10	0.376	0.369	0.372	0.372333	0.0020	51.21503897	48.78496103	
	3	0.728	0.725	0.722	0.725	0.0017	99.72489684	0.275103164	
	1	0.728	0.725	0.726	0.726333	0.0009	99.90829895	0.091701055	
CUR-Liposomes	300	0.019	0.019	0.018	0.018667	0.0003	2.567629528	97.43237047	15.49 ± 0.05
	100	0.026	0.024	0.022	0.024	0.0012	3.301237964	96.69876204	
	30	0.087	0.092	0.099	0.092667	0.0035	12.74644658	87.25355342	
	10	0.365	0.37	0.368	0.367667	0.0015	50.57313159	49.42686841	
	3	0.716	0.709	0.71	0.711667	0.0022	97.89087575	2.109124255	
	1	0.73	0.724	0.725	0.726333	0.0019	99.90829895	0.091701055	

## Conclusions

In this study, curcumin-loaded liposomes (CUR-liposomes) were successfully developed and characterized. The optimized formulation demonstrated enhanced anticancer activity across multiple human cancer cell lines while maintaining a safe profile in normal cells. These results highlight the potential of CUR-liposomes as an effective drug delivery system for cancer therapy. This plant-derived liposomal system represents a promising approach for enhancing curcumin’s therapeutic potential in cancer treatment. Future studies should focus on in vivo evaluation, long-term stability, and optimization of the liposomal formulation to further improve therapeutic efficacy and clinical applicability.

## Data Availability

No datasets were generated or analysed during the current study.

## Abbreviations

A549: Human lung adenocarcinoma epithelial cell line
Caco-2: Colon cancer cells
CUR: Curcumin
DLS: Dynamic light scattering
DOXO: Doxorubicin
DSC: Differential scanning calorimetry
FTIR: Fourier transform infrared
HPLC: High-performance liquid chromatography
IC50: Half maximal inhibitory concentration
MCF-7/ADR: Human multidrug-resistant breast adenocarcinoma cells
PANC-1: Pancreatic cancer cells
PC3: Human prostate cancer cells
TEM: Transmission electron microscopy

## References

[1] Siegel, R. L., Miller, K. D. & Jemal, A. Cancer statistics, 2020. *CA Cancer J. Clin.* **70**, 7–30 (2020). 31912902 10.3322/caac.21590

[2] Zhou, V. et al. The role of curcumin in cancer treatment. *Biomedicines* **9**, 1086 (2021). 34572272 10.3390/biomedicines9091086 PMC8464730

[3] Ravindran, J., Prasad, S. & Aggarwal, B. B. Curcumin and cancer cells: how many ways can curry kill tumor cells selectively? *AAPS J.* **11**, 495–510 (2009). 19590964 10.1208/s12248-009-9128-x PMC2758121

[4] Kunnumakkara, A. B. et al. Curcumin, the golden nutraceutical: multitargeting for multiple chronic diseases. *Br. J. Pharmacol.* **174**, 1325–1348 (2017). 27638428 10.1111/bph.13621 PMC5429333

[5] Teng, M., Hu, R. & Zhao, X. A review of curcumin and its derivatives as anticancer agents. *Int. J. Mol. Sci.* **20**, 1033 (2019). 30818786 10.3390/ijms20051033 PMC6429287

[6] Nakamura, K. et al. Discovery of a new function of curcumin which enhances its anticancer therapeutic potency. *Sci. Rep.* **6**, 30962 (2016). 27476814 10.1038/srep30962 PMC4967984

[7] Taira, M., Katagiri, K., Akiyama, R. & Yamada, K. Modulation of raft domains in a lipid bilayer by boundary-active curcumin. *Chem. Commun.* **50**, 10112–10115 (2014). 10.1039/c3cc47738j PMC3947710 24396862

[8] Komal, K., Chaudhary, S., Yadav, P., Pramanik, R. & Singh, M. The therapeutic and preventive efficacy of curcumin and its derivatives in esophageal cancer. *Asian Pac. J. Cancer Prev.* **20**, 1329–1336 (2019). 31127885 10.31557/APJCP.2019.20.5.1329 PMC6857884

[9] Fang, T., Wang, Y., Li, R. J. & Zhao, L. Liposomal curcumin and its application in cancer. *Int. J. Nanomed.* **12**, 6027–6044 (2017). 10.2147/IJN.S132434 PMC5573051 28860764

[10] Zheng, S. et al. Liposomal curcumin alters chemosensitivity of breast cancer cells to adriamycin via regulating MicroRNA expression. *Gene* **622**, 1–9 (2017). 28431975 10.1016/j.gene.2017.04.026

[11] Sharma, K. G., Kumar, K. & Gupta, A. Curcumin effect on cancer cells’ multidrug resistance: an update. *Phytother Res.* **34**, 2579–2591 (2020). 10.1002/ptr.6703 32307747

[12] Zhang, Y. Thin-film hydration followed by extrusion method for liposome preparation. *Methods Mol. Biol.* **1522**, 17–22 (2017). 27837527 10.1007/978-1-4939-6591-5_2

[13] Andra, V. V. S. N. L., Pammi, S. V. N., Bhatraju, L. V. K. P. & Ruddaraju, L. K. A comprehensive review on novel liposomal methodologies, commercial formulations, clinical trials and patents. *Bionanoscience* **12**, 731–748 (2022). 10.1007/s12668-022-00941-x PMC8790012 35096502

[14] Smith, B. C. *Fundamentals of Fourier Transform Infrared Spectroscopy* (CRC Press, 2011).

[15] Stetefeld, J., McKenna, S. A. & Patel, T. R. Dynamic light scattering: a practical guide and applications in biomedical sciences. *Biophys. Rev.* **8**, 409–427 (2016). 28510011 10.1007/s12551-016-0218-6 PMC5425802

[16] Priyadarsini, K. I. The chemistry of curcumin: from extraction to therapeutic agent. *Molecules* **19**, 20091–20112 (2014). 25470276 10.3390/molecules191220091 PMC6270789

[17] Tiernan, H., Byrne, B. & Kazarian, S. G. ATR-FTIR spectroscopy and spectroscopic imaging for the analysis of biopharmaceuticals. *Spectrochim Acta Mol. Biomol. Spectrosc.* **241**, 118636 (2020). 10.1016/j.saa.2020.118636 PMC7308041 32610215

[18] Yeo, S., Kim, M. J., Shim, Y. K., Yoon, I. & Lee, W. K. Solid lipid nanoparticles of curcumin designed for enhanced bioavailability and anticancer efficiency. *ACS Omega*. **7**, 36528–36539 (2022). 10.1021/acsomega.2c04407 PMC9558702 36249382

[19] Hasan, M. et al. Growth-inhibitory effect of chitosan-coated liposomes encapsulating curcumin on MCF-7 breast cancer cells. *Mar. Drugs*. **18**, 217 (2020). 32316578 10.3390/md18040217 PMC7230998

[20] Kumar, T., Ramesh, M., Li, M., Gao, Y. & Fang, Y. Stability and release performance of curcumin-loaded liposomes with varying content of hydrogenated phospholipids. *Food Chem.* **326**, 126973 (2020). 32413757 10.1016/j.foodchem.2020.126973

[21] Chen, X. et al. The stability, sustained release and cellular antioxidant activity of curcumin nanoliposomes. *Molecules* **20**, 14293–14311 (2015). 26251892 10.3390/molecules200814293 PMC6331986

[22] Lankalapalli, S. & Tenneti, V. Drug delivery through liposomes. In *Smart Drug Delivery* (eds. Tekade, R. K.) 1–24 (IntechOpen, 2022).

[23] Ahmed, S. A. et al. Development and characterization of soy lecithin liposome as potential drug carrier systems for doxorubicin. *J. Pharm. Innov.* **18**, 772–783 (2023).

[24] Le, N. T. T. et al. Soy lecithin-derived liposomal delivery systems: surface modification and current applications. *Int. J. Mol. Sci.* **20**, 4706 (2019). 31547569 10.3390/ijms20194706 PMC6801558

[25] Chen, Y. et al. Preparation of curcumin-loaded liposomes and evaluation of their skin permeation and pharmacodynamics. *Molecules* **17**, 5972–5987 (2012). 22609787 10.3390/molecules17055972 PMC6268695

[26] Ghoran, S. H. et al. Curcumin-based nanoformulations: a promising adjuvant towards cancer treatment. *Molecules* **27**, 5236 (2022). 36014474 10.3390/molecules27165236 PMC9414608

